# Psychometric properties, and cultural appropriateness, of patient reported outcome measures for use in primary healthcare: a scoping review

**DOI:** 10.1007/s11136-025-03956-5

**Published:** 2025-03-28

**Authors:** Christopher M. Doran, Jamie Bryant, Erika Langham, Roxanne Bainbridge, Anthony Shakeshaft, Breanne Hobden, Sara Farnbach, Megan Freund

**Affiliations:** 1 Cluster for Resilience and Wellbeing, Manna and Appleton Institutes, Central Queensland University, Level 4, 160 Ann Street, Brisbane, QLD 4000 Australia; 2 Health Behaviour Research Collaborative, School of Medicine and Public Health, College of Medicine and Wellbeing, University of Newcastle, Callaghan, NSW Australia; 3 Equity in Health and Wellbeing Program, Hunter Medical Research Institute, New South Wales, Australia; 4 Poche Centre, University of Queensland, St Lucia, QLD Australia; 5 National Drug and Alcohol Research Centre, UNSW, Sydney, NSW Australia

**Keywords:** Patient reported outcome measures, Review, Primary health care, COSMIN, Measurement properties, Outcome measurement instruments

## Abstract

**Purpose:**

To critically appraise the psychometric properties and cultural appropriateness of self‐reported generic patient-reported outcome measures (PROMs) applicable for use in the primary healthcare setting using the Consensus Based Standards for the Selection of Health Measurement Instruments (COSMIN) guidelines.

**Methods:**

PROMs were identified via a published systematic review and searches of relevant websites. PROMs were included if they were generic (i.e., outcome measures that assessed general aspects of health); had a maximum of 30 items; were applicable for use by all adult primary care patients; and were validated in English. Data was extracted regarding the characteristics of each PROM and the characteristics of included validation studies. The COSMIN risk of bias checklist was used to assess methodological quality and the revised COSMIN criteria was used to assess measurement properties. An evidence synthesis was conducted across studies using the guidelines from the modified Grading of Recommendations Assessment, Development and Evaluation approach for systematic reviews of clinical trials.

**Results:**

399 PROMs were identified and 19 met inclusion criteria. The included PROMs measured general health related quality of life (n = 8), outcomes or impact of care (n = 3), patient enablement, activation, and empowerment (n = 3), quality of care (n = 3), health and disability (n = 1), and functional status (n = 1). Six PROMs met the recommended COSMIN threshold for implementation.

**Conclusion:**

Although six PROMs can be recommended for use in primary care, further psychometric testing is still required to strengthen evidence related to internal consistency, responsiveness and cross-cultural validity/measurement invariance. Selection of a PROM for routine clinical use in primary care also needs to be guided by the patient population.

## Plain English summary

Psychometric properties, and cultural appropriateness, of patient reported outcome measures for use in primary healthcare: A scoping review. Patient-reported outcome measures (PROMs) are instruments used to report the status of a patient’s health condition from the patient’s perspective, without interpretation by a healthcare provider or others. This study aimed to understand the most appropriate PROM that is available for use in a primary health care setting. The availability of numerous PROMs creates challenges for clinicians and researchers in selecting the most appropriate one for their specific needs. Since primary care is the cornerstone of healthcare systems, selecting the most appropriate PROM is crucial to ensuring positive patient outcomes. This study found that only a limited number of PROMs can be recommended for use in primary care, with further work required to strengthen methodological quality and cultural appropriateness.

## Background

Patient-reported outcomes are defined as any report of the status of a patient’s health condition from the patient’s perspective, without interpretation by a healthcare provider or others [1, 2]. Patient-reported outcome measures (PROMs) are instruments used to capture subjective elements of patient views of their symptoms, functional status, and health related quality of life in a structured, standardised, and efficient way. PROMs can be generic (measure outcomes common across most patients accessing healthcare), disease-specific (applicable only to patients who have a certain disease) or population-specific (applicable only to patients with defined characteristics, for example children).

The fundamental importance of patient perspectives in ensuring the delivery of high-quality healthcare has increasingly been recognised by health‐care systems around the world, with the use of PROMs being touted as having the potential to change the way healthcare is organised and delivered [3]. PROMs can: contribute to the provision of care that is patient centered [4, 5]; assist the identification of health issues that may require further investigation or management; monitor changes in conditions and response to treatments over time; and promote shared decision making [6]. PROMs can also contribute to healthcare policy and decision making by providing information about the comparative effectiveness of treatments and for benchmarking and quality improvement activities, and can provide information about variations in care, costs, and outcomes among healthcare providers.

As a result of healthcare systems around the world increasingly adopting PROM use, there has been a considerable increase in the development of PROMs [7] covering a range of diseases, conditions, and contexts. A 2021 review that critically synthesised information on generic and selected condition‐specific PROMs to describe trends regarding their development and application in the period 1989–2019 identified 315 [8]. The number of available PROMs creates challenges for clinicians and researchers in selecting the most appropriate high-quality PROM for their specific needs. Selection of a PROM for use in clinical practice should consider if the PROM has been psychometrically validated to ensure it measures the latent construct intended, provides reliable responses, and can measure meaningful change. The feasibility of implementing the PROM, how the resultant data will be used, and suitability for the patient population are also important.

Primary care is the cornerstone of healthcare systems, often representing the first point of contact for individuals with the health system. The use of PROMs in primary care presents unique challenges. Patients attending primary care span the entire range of population age, and present with a wide range of diverse health and wellbeing conditions of varied complexity. In this setting, PROMs can have multiple uses from monitoring and informing the ongoing management of an individual’s health condition, through to informing ongoing quality improvement activities at both meso and macro levels [9]. In Australia, there are 31 Primary Health Networks funded by the Federal Government as local independent organisations tasked with ensuring that primary care in their defined regions is accessible, efficient and effective [10]. There is currently no recommended PROM for use in the primary care setting in Australia and no information relating to the potential cultural appropriateness of existing PROMs for Indigenous populations.

The aim of this research was to critically appraise the psychometric properties and cultural appropriateness of self‐reported generic PROMs applicable for use in the primary healthcare setting in Australia using the Consensus Based Standards for the Selection of Health Measurement Instruments (COSMIN) guidelines. This will provide essential evidence to guide the selection of the most appropriate high-quality PROM for use in primary care clinical practice.

## Methods

### Review advisory group

A Review Advisory Group was established to determine appropriate parameters for the review and ensure the evidence produced was useful to end users. The Review Advisory Group included 16 members, incorporating researchers with expertise in conducting systematic literature reviews and psychometric assessment, and end users with relevant knowledge and expertise. The Group met regularly to establish the scope and methodological approach for the review, refine inclusion and exclusion criteria, and provide advice and feedback about findings.

### Identification of potential PROMs for inclusion

A 2021 comprehensive review identified and critically synthesized information on generic and selected condition-specific PROMs [8], identifying 315 PROMs. This review searched the academic and grey literature to identify validated PROMs including searches of peer-reviewed databases, websites, Google Scholar, and Google searches. Given its exhaustiveness, the PROMs identified in the 2021 review were used as a starting point for identification of PROMs for current study, rather than conducting a repetitious literature search. To supplement the 315 PROMs previously identified and capture PROMs relevant to Australia, we also reviewed the generic PROMs available Australian Commission on Safety and Quality in Healthcare website (n = 40) [11], the PROMs available on the NSW Agency for Clinical Innovation website (n = 16) [12], the short-listed PROMs identified in an earlier systematic review of generic PROMs for primary care[13] (n = 20) and lists of PROMs provided that were currently being used in two organisations who collaborated on this review—Western Queensland Primary Health Network (https://www.wqphn.com.au/) (N = 7) and by Check-Up (https://checkup.org.au/ (N = 1).

### Inclusion and exclusion criteria

PROMs were included if they assessed general aspects of patient health, were patient reported, were applicable for use with adults aged 18 years and over and had content that was relevant for implementation in the primary care setting. As selection of PROMs for use in primary care (the focus of this review) requires consideration of the feasibility of implementation, therefore PROMs also needed to be < 30 Items OR have evidence of ability to complete the PROM in < 10 min. PROMs were excluded if they were disease, condition, or symptom specific; clinician or proxy reported; only relevant for sub-groups of patients with specific demographic characteristics (e.g., the elderly, veterans, children), or other characteristics (e.g., those taking over the counter medicine); or applicable only in non-primary care settings (e.g., in-patient or acute care settings).

Studies that used an eligible PROM needed to report on development and/or evaluation of one or more psychometric properties, be implemented in English, and the validation sample had to include primary care patients aged 18 years and older, patients with long term conditions, patients with chronic illness, or a general community sample. Studies that used an eligible PROM as an outcome measure, or studies that used the PROM as a comparator instrument in the validation study of another instrument, were excluded.

### Data extraction

For each PROM/study meeting the inclusion criteria, the following information was extracted by JB: purpose, target population, initial year of publication, country(ies) of development, mode(s) of administration, recall period, time to complete, readability, number of items, response options, domains, scoring, language and available translations, and licensing restrictions and costs. For each study reporting on psychometric properties, the following information was also extracted: description of participants (including relevant disease characteristics), participant details (number of participants, gender, and age), instrument administration (including setting and method of recruitment, country, and language) and overall response rate.

### Assessment of methodological quality and psychometric characteristics

Two reviewers independently undertook all data coding, with discrepancies discussed to achieve consensus about ratings.

#### Appraisal of study methodological quality

The methodological quality of included studies were assessed using the Consensus-based Standards for the selection of health status Measurement Instruments (COSMIN) risk of bias checklist [14, 15], a tool developed specifically for use in systematic reviews of PROMs. Ten properties of quality were assessed regarding the development or validation of a PROM: development, content validity, structural validity, internal consistency, cross-cultural validity/measurement invariance, reliability, measurement error, criterion validity, hypotheses testing for construct validity, and responsiveness. Each property was rated on a five-point scale as “very good,” “adequate”, “doubtful”, and “inadequate”, or “not assessed”. The overall score for each measurement property was decided using a ‘worst score counts’ approach (e.g., if four out of the five criteria assessed for structural validity were rated as ‘very good’, but one was rated ‘inadequate’, the score given for the entire quality property was ‘inadequate’).

Modified criteria were used to assess the methodological quality of PROM development and content validity due to the stringent COSMIN checklist quality threshold requirements. These modifications were required due to time and resource constraints. PROM development was restricted to assessment of ‘general design requirements. Assessment of content validity was restricted to assessment of whether an appropriate method was used to ask patients about the relevance of the items to their experience of the condition and whether an appropriate method was used to ask professionals whether each item is relevant for the construct of interest. Content validity was assessed globally (i.e., one rating per PROM rather than per study), and included assessment of multiple documents in addition to cited studies (e.g., PROM user manuals, other papers reporting solely on PROM development). As per COSMIN guidelines, instruments that had been modified (e.g., shortened) were evaluated as a new instrument. However, for these PROMs, previously conducted development and content validity studies were considered as relevant for the rating for PROM development and content validity.

#### Appraisal of PROM measurement properties

The measurement properties of each PROM were assessed using a revised version of the COSMIN criteria for good measurement properties that had been developed with reference to the criteria included in the COSMIN user manual [15] and used in two previous reviews [16, 17]. The revised checklist simplifies some of the stringent assessments required in the original COSMIN checklist, particularly related to assessment of content validity. Nine measurement properties were assessed for each included study: content validity, structural validity, internal consistency, cross-cultural validity and measurement invariance, reliability, measurement error, criterion validity, hypotheses testing for construct validity, and responsiveness. The criteria outlined in Table 2 were applied, with ratings of sufficient ( +), insufficient ( −) or indeterminate (?) provided for each psychometric property within each study.

### Evidence synthesis

Following the appraisal of methodological quality and measurement properties for each study, an evidence synthesis was conducted for each PROM. First, we determined the overall evidence for each measurement property, categorising it as sufficient (+), insufficient (−), inconsistent (±), or indeterminate (?). A PROM was deemed to have sufficient or insufficient overall quality if more than 50% of individual studies were rated the same way; otherwise, it was rated as inconsistent. Next, we graded the quality of evidence for each measurement property based on the modified Grading of Recommendations Assessment, Development and Evaluation (GRADE) approach. Each property was initially rated as high quality, with downgrades applied for risk of bias (inadequate or doubtful methodological quality), inconsistency (unexplained variability in results across studies), and indirectness (evidence derived from populations different from the target population). Imprecision was not considered a downgrade criterion since the total sample size across all studies exceeded 200. Additionally, no quality rating was assigned when a measurement property was classified as indeterminate.

As per the COSMIN guidelines [18], PROMs were considered Class A PROMs and able to be recommended for use if they had evidence for sufficient content validity (any level) AND at least low quality evidence for sufficient internal consistency. A PROM was considered a class C PROM and not able to be recommended for use if it had high quality evidence for an insufficient measurement property. Remaining PROMs were classified as Class B PROMS with potential to be recommended for use but require further research to assess quality.

## Results

### PROM identification

A total of 399 PROMs were identified and following removal of duplicates, 338 POMS were evaluated against inclusion criteria with 319 excluded for the following reasons disease or condition specific (n = 301), not self-reported (n = 2), not relevant for primary care setting (n = 15) or population specific (n = 1). Nineteen PROMs met inclusion criteria and were included in the review (see Table 1). Three PROMs had multiple versions available: the EQ-5D has both a 3 Level (EQ-5D-3L) and 5 Level (EQ-5D-5L) version; there were two versions of the Patient Activation Measure (a 13 item and 22 item version) and there were three versions of Patient Assessment of Chronic Illness Care (a 20 item, 13 item and 14 item version). Detailed characteristics of the PROMs and characteristics of the validation papers (study population and implementation) are outlined in the supplementary material.

**Table 1:** Summary of included PROMs by category (n = 19)

	PROM name	Abbreviations
Health-related quality of life	EuroQol-5 Dimension HowRU Patient reported outcome measurement system-29 Patient reported outcome measurement system-Global Health Short Form health survey-12 World Health Organisation quality of life Measure Yourself Medical Outcomes Profile	EQ-5D-3L, EQ-5D-5L HowRU PROMIS-29 PROMIS-10 SF-12 WHOQoL-Brief MYMOP
Enablement, activation, empowerment	Patient Activation Measure Patient Enablement Instrument	PAM-13, PAM-22 PEI
Outcomes or impact of care	Long term conditions questionnaire Outcomes related to Impact on Daily Living Primary care outcomes questionnaire	LTCQ ORIDL PCOQ
Functional status	Dartmouth COOP/COOP WONCA Charts	COOP
Health and disability	World Health Organisation Disability Assessment Schedule	WHODAS
Quality of care	Patient Assessment of Chronic Illness Care	PACIC-20, PACIC-13, PACIC-14

### Key characteristics of eligible PROMs

Table 1 provides a summary of the key concepts measured by each PROM. Eight PROMs measured general health related quality of life, three measured enablement, activation, and/or empowerment, three measured outcomes or impact of care, and three measures quality of care. One PROM each examined functional status and health and disability.

#### Countries of development

Six PROMs were developed in the UK (HowRU, LTCQ, MYMOP, ORIDL, PEI, PCOQ), nine were developed in the USA (COOP, PAM-13/22 PACIC-13/14/20, PROMIS-29, PROMIS-10, SF-12) and four were developed across multiple countries (WHOQoL-BREF, WHO DA, EQ-5D-3L, EQ-5D-5L).

#### Target populations

Six PROMs were specifically developed for use in primary care (COOP, HowRU, MYMOP, ORIDL, PEI, PCOQ). Seven generic PROMs were developed for application across settings (EQ-5D-3L, EQ-5D-5L, PROMIS-29, PROMIS-10, SF-12, WHODAS, WHOQoLBrief) and six were developed to assess patient reported outcomes in people with long term or chronic conditions (LTCQ, PAM-13/22, PACIC-13/14/20).

#### Mode of administration, length and completion time

Most PROMs are administered as pen and paper surveys (n = 13). Six PROMs can be administered as a pen-and-paper survey or through interviewer administration (face-to-face or telephone). The PROMIS-29 and PROMIS-10 Global Health can be administered via pen and paper or electronically, while the PAM-13, PAM 22, EQ-5D-3L, EQ-5D-5L and SF-12 can be administered via pen and paper, interviewer or electronically. The shortest measures were the HowRU and MYMOP which each contained only 4 questions, and both used pictorial response scales.

#### Readability

Reading age was reported for three PROMs. The HowRU, which was the shortest included PROM, had the highest readability level with a Flesch-Kincaid Grade Level of 1.9 and a reading ease of 89 [19]. The EQ-5D-3L was reported to have “very easy” to “fairly easy” readability and a Flesch-Kincaid grade level of 4.2 [20]. The PAM-13 had the lowest readability level of the PROMs that reported this, with a Flesch-Kinkaid grade level of 8.2, and a reading age of 13 in a general population sample.

### Methodological quality

Table 2 presents COSMIN risk of bias ratings for the methodological quality for the included studies reporting the psychometric properties of the 19 PROMs (very good, adequate, doubtful and inadequate). Most PROMs were rated as very good or adequate for development, with clear descriptions provided of the construct to be measured, the theoretical or conceptual origin of the construct, and the target population for which the PROM was developed. The MYMOP was rated as inadequate as the construct being measured was described only as a patient generated measure that evaluated symptoms and activity of daily living, and the sample used for development was not described. Content validity was rated as very good or adequate for nine PROMs, but inadequate for four PROMs (COOP Charts, HowRU, Measure Yourself Outcomes Profile and Outcomes related to Impact on Daily Living) because of lack of clear involvement of patients and/or professionals in item development. Of the remaining properties, the most frequently evaluated were construct validity (27 studies), structural validity (20 studies) and internal consistency (17 studies). Criterion validity was not rated for most studies due to lack of a ‘gold standard’ comparison for the included measures, according to the COSMIN definition [15]. The only exceptions were comparisons of the SF-12 with the SF-36, and the EQ-5D-5L with the EQ-5D-3L. Four studies reported cross cultural validity/measurement invariance, and two reported measurement error. The three included studies that reported the psychometric properties of the PAM-13/22 provided the most evidence for psychometric testing, able to be rated on eight out of ten properties.

**Table 2:** Summary of methodological quality (very good/adequate/doubtful/inadequate) and measurement properties (+ , −, ?^b^) for each paper reporting on a shortlisted PROM (n = 19 PROMs reported in n = 32 studies)

PROM name	Prom development	Content validity	Structural validity	Internal consistency	Cross cultural/measurement invariance	Reliability	Measurement error	Criterion validity	Construct validity	Responsiveness
*COOP charts* Jenkinson [32]-9 item Kinnersley [30]-6 item Nelson [33]-9 item	Very good	Inadequate/?	0 0 0	0 0 0	0 0 0	0 Inadequate/? Doubtful/−	0 0 0	0 0 0	Adequate/− Doubtful/+ Very good/+	0 Doubtful/+ 0
*EQ-5D* Johnson [34]-3L Jannsen [35]-3L Zakershrak [36]-3L Santiago [37]-5L Jannsen [35]-5L	Very good	Adequate/+	0 0 Very good/+ Very good/+ 0	0 0 0 0 0	0 0 0 0 0	0 0 0 0 0	0 0 0 0 0	0 0 Very good/+ 0	Adequate/+ Adequate/− 0 Adequate/+ Adequate/−	0 0 0 0 0
*HowRU* Benson [19]	Adequate	Inadequate/?	Adequate/+	Very good/+	0	0	0	0	Very good/+	0
*Long-term conditions questionnaire* Potter [27] Batchelder [25]	Very good	Very good/+	Adequate/+ Very good/+	Very good/+ Very good/+	0 Very good/−	Very good/+ 0	0 0	0 0	Very good/+ Very good/+	0 0
*Measure yourself outcomes profile* Paterson [38]	Inadequate	Inadequate/?	0	0	0	0	0	0	Very good/−	Doubtful/?
*Outcomes related to impact on daily living* Reilly [39]	Doubtful	Inadequate/?	0	0	0	0	0	0	0	Very good/−
*Patient activation measure* Hibbard 2004 20-item [28] Hibbard 2005 13-item [40] Hung 13 item [26]	Very good	Very good/+	Very good/+ Very good/+ Very good/+	Very good/+ 0 Very good/+	0 0 Adequate/−	Adequate/+ 0 0	Adequate/+ Adequate/+ 0	0 0 0	Very good/+ Very good/+ Inadequate/−	Very good/? 0 0
*Patient assessment of chronic illness care* Glasgow [41]-20 item Taggart [42]-20 item Rick [43]-20 item Lambert [21]-14 item Gibbons [22]-13 item	Very good	Doubtful/−	Very good/+ Very good/+ Very good/− Very good/+ Very good/+	Very good/+ Very good/+ Very good/− Very good/− 0	0 0 0 Inadequate/− 0	Inadequate/− 0 0 0 Very good/+	0 0 0 0 0	0 0 0 0 0	Inadequate/+ Very good/? Inadequate/− 0 0	0 0 0 0 0
*Patient enablement instrument* Howie 1997 [44] Howie 1998 [45] Mead [46]	Very good	Doubtful/−	0 0 0	Very good/+ Very good/+ 0	0 0 0	0 0 0	0 0 0	0 0 0	Inadequate/− Very good/+ Adequate/?	0 0 0
*PROMIS-29* Hays [47] Fischer [48] Cella	Very good	Very good/+	Very good/+ Very good/+ 0	Very good/+ Very good/+ 0	0 Very good/+ 0	0 0 0	0 0 0	0 0 Very good/+	Very good/+ Very good/+ Adequate/+	0 0 0
*PROMIS-10 global health* Hays[49]	Very good	Very good/+	Very good/−	0	0	0	0	0	Very good/+	0
*Primary care outcomes questionnaire* Murphy [31]	Very good	Very good/+	Adequate/+	Very good/+	0	0	0	0	Adequate/+	Inadequate/+
*SF-12* Ware [29] Cheak-Zamora [50]	Very good	Adequate/+	0 0	0 Very good/+	0 0	Doubtful/+ Very good/−	0 0	Inadequate/+ 0	Very good/− Doubtful/−	0 0
*WHODAS* Andrews [51] Rhem [52]	Very good	Very good/+	Very good/+ Very good/+	0 Very good/+	0 0	0 0	0 0	0	0 Inadequate/?	0 0
*WHOQoL-brief* WHOQoL group [23] Skevington [24]	Very good	Very good/+	Very good/? Adequate/+	Very good/− Very good/−	0 Very good/?	Doubtful/− 0	0 0	0 0	Adequate/+ Adequate/?	0 0

0 = property not assessed

^a^Methodological quality was rated for each study as very good, adequate, doubtful or inadequate in line with definitions provided by the Consensus-based Standards for the selection of health status Measurement Instruments (COSMIN) risk of bias checklist

^b^Measurement properties were rated using a revised version of the COSMIN criteria for good measurement properties using ratings of sufficient (+), insufficient (−) or indeterminate (?)

### Measurement properties

Table 2 provides the COSMIN ratings for measurement properties for the included studies reporting the psychometric properties of the 15 PROMs (sufficient (+), insufficient (−) or indeterminate (?)). Nine PROMs had sufficient content validity (EQ-5D-3L/EQ-5D-5L, Long-term Conditions Questionnaire, PAM-13/22, PROMIS-29, PROMIS-10, Primary Care Outcomes Questionnaire, SF-12, WHODAS and WHOQoL- Brief). Four PROMs had indeterminate ratings as not enough information was provided to rate content validity (COOP Charts, HowRU, Measure Yourself Outcomes Profile, Outcomes related to Impact on Daily Living), and two PROMs had insufficient content validity as there was no clear involvement of patients and/or professionals in development of tool (Patient Assessment of Chronic Illness Care, Patient Enablement Instrument). Structural validity was sufficient for 17 of the 20 studies that examined this, except for the Patient Assessment of Chronic Illness tool where one study found insufficient evidence of structural validity for the 20-item version, and PROMIS-10. Twelve of the sixteen studies that reported internal consistency were rated as sufficient. Two studies reporting on the internal consistency of the PACIC-14 and PACIC-20 and two studies reporting on the internal consistency of the WHOQOL-BREF were rated as insufficient as Cronbach alphas did not meet threshold scores [21–24]. Cross cultural validity/measurement invariance was rated as sufficient for the PROMIS-29, indeterminate for the WHOQoL-brief, and insufficient in the remaining three studies measuring this [21, 25, 26] as a result of differential item functioning being found for items. Evidence for sufficient test–retest reliability was found for four studies [22, 27–29]. Construct validity was evaluated in 27 studies and was rated as sufficient in 15 of these. Only two studies reporting on the COOP charts [30] and the Primary Outcomes Questionnaire [31] demonstrated evidence of responsiveness.

### Evidence synthesis

GRADE outcomes for PROMs overall, together with the overall ratings for measurement properties, are provided in Table 3. Six PROMs had evidence for sufficient content validity and at least low-quality evidence for sufficient internal consistency, meeting the COSMIN threshold as being able to be recommended for implementation. These were: the Patient Activation Measure (measuring patient activation and empowerment), PROMIS-29 and SF-12 (measuring health related quality of life), the Primary Care Outcomes Questionnaire and Long-term Conditions Questionnaire (measuring outcomes or impact of care), and the WHODAS (measuring health and disability). Seven PROMs (COOP Charts, HowRU, Measure Yourself Outcomes Profile, Outcomes related to Impact on Daily Living, Patient Assessment of Chronic Illness Care, Patient Enablement Instrument, WHOQoL-Brief) had evidence for insufficient or indeterminate content validity or insufficient internal consistency and were classified as Class C PROMs. The remaining two PROMs, the EQ-5D PROMIS-10, require further validity testing before they can be recommended for use.

**Table 3:** Quality of evidence overall for PROMs (n = 19)

PROM name	Structural validity	Internal consistency	Cross cultural/measurement invariance	Reliability	Measurement error	Criterion	Construct	Responsiveness	Overall rating
COOP charts	0	0	0	Very low/ ±	0	0	Low/+	Low/+	C
*EQ-5D* 3L 5L	High/+ High/+	0 0	0 0	0 0	0 0	High/+ 0	Moderate/ ± Moderate/ ±	0 0	B B
HowRU	Moderate/+	Moderate/+	0	0	0	0	High/+	0	C
Long-term conditions questionnaire	High/+	High/+	High/−	High/+	0	0	High/+	0	A
Measure yourself outcomes profile	0	0	0	0	0	0	High/−	?	C
Outcomes related to impact on daily living	0	0	0	0	0	0	0	High/−	C
*Patient activation measure* PAM-22 PAM-13	High/+ High/+	High/+ High/+	0 Moderate/−	Moderate/+ 0	Moderate/+ Moderate/+	0 0	High/+ Moderate/ ±	? 0	A A
*Patient assessment of chronic illness care* PACIC-20 PACIC-13 PACIC-14	High/ ± High/+ High/+	High/ ± 0 High/−	0 0 Very low/−	Very low/− High + 0	0 0 0	0 0 0	Very low/ ± 0 0	0 0 0	C C C
Patient enablement instrument	0	0	0	0	0	0	Moderate/ ±	0	C
PROMIS-29	High/+	High/+	High/+	0	0	High/+	High/+	0	A
PROMIS-10 global health	High/−	0	0	0	0	0	High/+	0	B
Primary care outcomes questionnaire	Moderate/+	Moderate/+	0	0	0	0	Moderate/+	Very low/+	A
SF-12	0	0	0	Moderate/ ±	0	Very low/+	Moderate/−	0	B
WHODAS	High/+	High/+	0	0	0	0	?	0	A
WHOQoL-brief	Moderate/+	Moderate/−	?	Low/−	0	0	Moderate/+	0	C

Each measurement property for a PROM was rated as having overall sufficient (+), insufficient (−), inconsistent (±) or indeterminate (0) evidence. Quality of evidence for each measurement property of each PROM was rated as high, moderate, low, or very low based on the guidelines from the modified Grading of Recommendations Assessment, Development and Evaluation (GRADE) approach [18]. In line with COSMIN guidelines, internal consistency could not be rated higher in quality that structural validity. Internal consistency was only rated where structural validity was established

## Discussion

This review critically appraised the psychometric properties of self‐reported generic PROMs applicable for routine clinical use in the primary healthcare setting in Australia. Of nineteen PROMs included, six had evidence of sufficient content validity and internal consistency per the COSMIN criteria and can be recommended for use in primary care. However, our findings highlight significant gaps in evidence about the psychometric properties of PROMs relevant for the primary care setting. No PROMs had evidence of psychometric validity on all ten criteria examined. For five PROMs, there was evidence of the psychometric properties from only a single validation study and for four PROMS there was evidence from only two validation studies. Cross-cultural validity/measurement invariance, responsiveness, and criterion validity have been assessed for very few PROMs, and only 6 PROMs had evidence of reliability. Failure to undertake robust psychometric evaluation may be unsurprising given the cost and time-consuming nature of scale development and the large sample sizes that are required. Nonetheless, given the increasing implementation of PROMs in healthcare as a means of achieving patient-centred care, it is critical that psychometrically sounds tools are available.

Although six PROMs met COSMIN criteria as Class A PROMs (recommended for use), further psychometric testing of these PROMs is still required. Responsiveness over time of the PAM-22 and PAM-13 needs to be established given some evidence of ceiling effects in other contexts [53–55], as does differential item functioning for the PAM-22 [53]. Additional work is also needed to explore whether refinement would resolve differential item functioning for the PAM-13 [26]. The Long-term Conditions Questionnaire requires evidence for cross-cultural validity/measurement invariance and the minimally important clinical difference for Primary Care Outcomes Questionnaire change scores are not yet established. Four PROMs, the EQ-5D-3L, EQ-5D-5L, PROMIS-10 and SF-12, were designated Class B PROMs that require additional psychometric testing before they can be recommended for use. While widely used, the EQ-5D-3L, EQ-5D-5L, PROMIS-10 and SF-12 did not have any evidence for internal consistency. Establishing the internal consistency of these PROMs in the primary care setting is needed before they can be recommended for use. Healthcare providers selecting PROMs for use in primary care should consider these findings when choosing the most appropriate tools for their patient populations. Recommendations for healthcare providers include prioritising PROMs with strong psychometric evidence, such as those classified as Class A, while acknowledging the need for further validation of these tools in the primary care context. Caution should be exercised in using the EQ-5D-3L, EQ-5D-5L, PROMIS-10 and SF-12 in primary care until internal consistency is confirmed in primary care populations.

Challenges in implementing PROMs in primary care include the feasibility of use with the patient population. Other research also supports the feasibility of PROMs in the Australian healthcare context. In 2014/15, a pilot study evaluated implementation of the PAM-13 [56] with a convenience sample of 1490 people. It found that 97% of participants completed the PAM-13 within 5–10 min and 93% indicated no difficulty in answering questions. The PROMIS-29 is currently used in the public health sector in NSW to measure patient reported outcomes in hospitals. Additionally, how the captured data will be used should be carefully considered in deciding. A significant advantage of the SF-12 is that it is one of the few PROMs that can be used to measure both health-related quality of life and health state utility value.

Despite different ways of conceptualising health among Indigenous populations, we identified no PROMs developed specifically for use with Indigenous populations and only one study included in the review examined the psychometric properties of a PROM (the EQ-5D) with an Aboriginal and/or Torres Strait Islander sample. Perceptions of health, wellbeing and what is an important outcome of healthcare varies across cultures [57]. While work is currently underway by the New South Wales (NSW) Agency of Clinical Innovation to examine the cultural appropriateness and validity of the PROMIS-29 for assessing health outcomes amongst Aboriginal and Torres Strait Islander people with diabetes in NSW [58], the use of existing general PROMs may not be suitable given most are underpinned by a western cultural perspective and traditional biomedical perceptions of health and wellbeing [57, 59]. There is a need for further work to establish a culturally appropriate and meaningful PROM for use with Aboriginal and Torres Strait Islander people in Australia. A 2019 systematic review identified domains of wellbeing relevant to Aboriginal and Torres Strait Islander Australians for measuring quality of life [60]. These included autonomy, empowerment and recognition; family and community; culture, spirituality and identity; Country; basic needs; work, roles, and responsibilities; education; physical health; and mental health. These identified domains are important to consider in any future work developing an Aboriginal and/or Torres Strait Islander specific PROM. PROMs should be developed with Indigenous knowledges, methodologies and methods [59].

### Strengths and limitations

This review provides a detailed summary of the methodological and measurement properties of PROMs available to assess patient reported outcomes in primary health care. While rigorous COSMIN methodology was utilised to undertake the review and make recommendations, the findings should be considered with regard to several limitations. Recommendations about suitable PROMs needed to be provided in a short time frame, which limited the feasibility of undertaking a primary review of the literature. As a result, potentially eligible PROMs were identified from an existing recently published review that used a comprehensive search of the academic and grey literature, supplemented by our own searches. This pragmatic approach may have led to the exclusion of some recently published PROMs that were not identified in the existing review. Additionally, the reliance on secondary data introduces the possibility of overlooking relevant studies or variations in PROM performance missed in the first review, which could have been captured through a more exhaustive primary review process. Finally, studies were restricted to those that implemented English language versions of PROMs. While psychometric performance may differ by language and across cultures [61], this limits the generalisability of our findings to non-English speaking populations. Additional research is needed to assess the psychometric properties of PROMs published in languages other than English, which would provide a more comprehensive understanding of their cross-cultural applicability and performance.

## Conclusions

The use of psychometrically sound and culturally appropriate PROMs in primary care is essential for achieving patient-centred care. Six PROMs measuring patient activation and empowerment, health related quality of life, outcomes of care, and health and disability met COSMIN criteria and can be recommended for use in primary care, although all require additional psychometric testing. These findings emphasise the need for future work to develop high quality validated PROMs that can be integrated into routine clinical practice across primary care settings. Selecting a PROM for implementation in primary care requires consideration of psychometric properties as well as feasibility and patient relevance. Further work is needed to develop a culturally appropriate and meaningful PROM for use with Aboriginal and Torres Strait Islander people in Australia that resonates with their unique health experiences, cultural values, and worldviews. Policymakers and healthcare providers should prioritise supporting the development, validation, and integration of PROMs to improve patient outcomes and care delivery.

## Data Availability

Data sharing is not applicable to this article as no datasets were generated or analysed during the current study.
